# Immediate Root-Filling

**Published:** 1887-11

**Authors:** 


### IMMEDIATE ROOT-FILLING.

In dentistry, as in medicine, the constant tendency is toward the exaggerated employment of a commendable practice or remedy. When microbes were first demonstrated to be the cause of certain zymotic disorders, many physicians, possessed of more enthusiasm than judgment, straightway jumped to the conclusion that the origin of all diseases was now discovered, and antiseptics was the specific for everything, from corns to consumption. "Gaseous enemata" is the latest exploded method, but for a time every tuberculous patient had a tube in his fundament.

In dentistry the latest craze is immediate root-filling. That dentists in the past have medicated too much cannot be successfully disputed, but it seems to us that we are now rushing to the other extreme. That in root-canals, long septic, it is sufficient to introduce a disinfectant, this to be immediately followed by a germicide and that by a permanent root-filling, does not seem to us like a safe practice. The products of infection are frequently infiltrated into the tissues, not only of the tooth itself, but of the territory beyond it, and it takes time for the agents to reach the most distant point. It is impossible in many instances to know when the whole is made entirely aseptic.

Again, there may be constitutional disturbances which demand prolonged treatment, in which the healing process must be begun before it is safe to leave nature to her unaided resources. There are products which must be absorbed or otherwise disposed of. There is a low chronic stage of disease, with but slight recuperative powers in the system to commence and carry on the repairs demanded. What surgeon dares close up a deep cavity without providing means for drainage, if the wound be reinfected ?

A root cannot be well filled until it is entirely dry, and there is frequently an effusion of mositure, of one character or another, which lasts for some time before it can be entirely checked. There are other reasons which may forbid immediate root-filling, yet we are convinced that it might with profit be practiced much oftener than it now is. But enthusiastic speakers in the heat of debate sometimes make exaggerated statements, forgetting the forbidding circumstances, and urging as without exceptions a rule which is only applicable in a majority of cases, while inexperienced young listeners seize upon the declaration, perhaps but partially comprehended, and run off with the shell upon their backs, like half-hatched chickens, only to meet with disappointment and to curse the day in which they attended a society meeting.

Such a case recently came under our own observation, and was a corollary to the debate upon root-filling at the late meeting of the American Dental Association. We do not think that the dentist who made the mistake in question, and who certainly is excelled in intelligence by very few, will again fill a canal which has long been septic until he is more sure of his ground and has tested the conditions. Nor will he again place implicit confidence in the dogmatic assertion of any man until he has tried the matter for himself. "Prove all things; hold fast to that which is good," is an excellent rule of conduct in every situation.

